# Supplementary material for: HIVprotI: an integrated web based platform for prediction and design of HIV proteins inhibitors
Source: J Cheminform. 2018 Mar 9;10:12. doi: 10.1186/s13321-018-0266-y (PMC5845081; doi:10.1186/s13321-018-0266-y)
Supplement: Supplementary file 2 — Additional file 2. Source code of HIVProtI web server. [file 13321_2018_266_MOESM2_ESM.zip › HIVprotI_Source-code/hivprotI/weka-3-6-6/doc/weka/gui/treevisualizer/TreeDisplayEvent.html]

TreeDisplayEvent


---


|  |  |  |
| --- | --- | --- |
| **Overview** \| **Package** \| **Class** \| **Tree** \| **Deprecated** \| **Index** \| **Help** \| **Weka's home** | |  |
| **PREV CLASS**   **NEXT CLASS** | **FRAMES**    **NO FRAMES**     **All Classes** |  |
| SUMMARY: NESTED \| FIELD \| CONSTR \| METHOD | DETAIL: FIELD \| CONSTR \| METHOD |  |


---


## weka.gui.treevisualizer Class TreeDisplayEvent

```
java.lang.Object
  weka.gui.treevisualizer.TreeDisplayEvent
```

---

``` public class TreeDisplayEvent extends java.lang.Object ```

An event containing the user selection from the tree display

**Version:**
:   $Revision: 7059 $

**Author:**
:   Malcolm Ware (mfw4@cs.waikato.ac.nz)

---

| **Field Summary** | |
| --- | --- |
| `static int` | `ACCEPT`             States that the user has accepted the tree. |
| `static int` | `ADD_CHILDREN` |
| `static int` | `CLASSIFY_CHILD`             Asks for another learning scheme to classify this node. |
| `static int` | `NO_COMMAND` |
| `static int` | `REMOVE_CHILDREN` |
| `static int` | `SEND_INSTANCES`             Command to remove instances from this node and send them to the VisualizePanel. |


**Constructor Summary**

`TreeDisplayEvent(int ar,
java.lang.String id)`
  
          Constructs an event with the specified command
and what the command is applied to.


| **Method Summary** | |
| --- | --- |
| `int` | `getCommand()` |
| `java.lang.String` | `getID()` |

**Methods inherited from class java.lang.Object**

`equals, getClass, hashCode, notify, notifyAll, toString, wait, wait, wait`

**Field Detail**

### NO\_COMMAND

```
public static final int NO_COMMAND
```

**See Also:**: Constant Field Values

---


### ADD\_CHILDREN

```
public static final int ADD_CHILDREN
```

**See Also:**: Constant Field Values

---


### REMOVE\_CHILDREN

```
public static final int REMOVE_CHILDREN
```

**See Also:**: Constant Field Values

---


### ACCEPT

```
public static final int ACCEPT
```

:   States that the user has accepted the tree.

    **See Also:**: Constant Field Values

---


### CLASSIFY\_CHILD

```
public static final int CLASSIFY_CHILD
```

:   Asks for another learning scheme to classify this node.

    **See Also:**: Constant Field Values

---


### SEND\_INSTANCES

```
public static final int SEND_INSTANCES
```

:   Command to remove instances from this node and send them to the
    VisualizePanel.

    **See Also:**: Constant Field Values


**Constructor Detail**

### TreeDisplayEvent

```
public TreeDisplayEvent(int ar,
                        java.lang.String id)
```

:   Constructs an event with the specified command
    and what the command is applied to.

    **Parameters:**: `ar` - The event type.: `id` - The id string for the node to perform the action on.


**Method Detail**

### getCommand

```
public int getCommand()
```

:   **Returns:**: The command.

---


### getID

```
public java.lang.String getID()
```

:   **Returns:**: The id of the node.


---


|  |  |  |
| --- | --- | --- |
| **Overview** \| **Package** \| **Class** \| **Tree** \| **Deprecated** \| **Index** \| **Help** \| **Weka's home** | |  |
| **PREV CLASS**   **NEXT CLASS** | **FRAMES**    **NO FRAMES**     **All Classes** |  |
| SUMMARY: NESTED \| FIELD \| CONSTR \| METHOD | DETAIL: FIELD \| CONSTR \| METHOD |  |


---
